# Analytical Performance of the New Sysmex High-Sensitivity Troponin T Assay

**DOI:** 10.3390/diagnostics15151838

**Published:** 2025-07-22

**Authors:** Chin Shern Lau, Napthathorn Asavapuriyothin, Chee Hoe Low, Soon Kieng Phua, Yali Liang, Tar Choon Aw

**Affiliations:** 1 Department of Laboratory Medicine, Changi General Hospital, Singapore 529889, Singapore; soon_kieng_phua@cgh.com.sg (S.K.P.); yali_liang@cgh.com.sg (Y.L.); tarchoon@gmail.com (T.C.A.); 2 Sysmex Asia-Pacific, Singapore 528735, Singapore; naphathorn@sysmex.co.th (N.A.); low.cheehoe@sysmex-ap.com (C.H.L.); 3 Department of Medicine, Yong Loo Lin School of Medicine, Singapore 119077, Singapore; 4 Academic Pathology Program, Duke-NUS Graduate School of Medicine, Singapore 169857, Singapore

**Keywords:** troponins, immunoassay, acute coronary syndrome

## Abstract

**Background:** We evaluated the Sysmex Highly Integrated Single-Cartridge Luminescence Immunoassay System (HISCL) hs-cTnT assay, and compared its performance to the Roche assay, with derivation of 99th-percentile upper reference limits (99% URLs) for healthy subjects. We assessed the effect of increasing age/decreasing eGFR on the HISCL hs-cTnT. **Methods:** We verified assay limits of blank/detection, precision and the functional sensitivity. Samples were analyzed on both the Sysmex HISCL and Roche Elecsys analyzers for method comparison. **Results:** The HISCL assay limit of blank/detection was 1.3/1.9 ng/L, and concentrations corresponding to 20/10% CVs were 1.8/3.3 ng/L. Assay precision of kit controls at 3253 ng/L was 2.2% and at 106 ng/L was 2.5%. Linear regression analysis (*n* = 2151) showed good agreement (r = 0.95) with the Roche hs-cTnT. Bland–Altman (Roche/HISCL) analysis for samples with hs-cTnT ≤ 52 ng/L showed a mean absolute difference of 3.5 ng/L; for hs-cTnT > 52 ng/L, the mean difference was 2.8%. In a cardio-renal healthy population (*n* = 1004), the 99% URLs were 14.4/17.0/13.9 ng/L for overall/male/female, respectively; assay CV% was below 10% at these levels. More than 50% of the hs-cTnT in the healthy male and female subjects were measurable above the limit of detection. Hs-cTnT increased with increasing age and decreasing eGFR. **Conclusions:** In conclusion, the Sysmex HISCL hs-cTnT fulfils the criteria for a high-sensitivity assay, with specific 99th URLs for males and females. Expectedly, the baseline Sysmex hs-cTnT increases with age and decreasing eGFR.

## 1. Introduction

Cardiac troponins remain an essential tool in the diagnosis of acute coronary syndromes (ACS). The European Society of Cardiology (ESC) have recommended the use of 0/1-h or 0/2-h troponin guidelines to quickly rule-in/out ACS since 2023 [1]. They have been used to great effect, with high negative predictive values for 30-day cardiac death or MI (up to 96.6/98.9% among patients with/without known coronary artery disease) [2]. In addition, high-sensitivity cardiac troponins have also proven themselves as important prognostic factors in cardiac disease, with higher levels associated with adverse cardiac outcomes, non-cardiovascular disease-related death, heart failure, stroke and mortality [3]. According the Fourth Universal Definition of Myocardial Infarction [4], a cut-off point of greater than the 99th-percentile upper reference limit (99% URL) of the general population can be used to differentiate patients with myocardial injury from those without. Additionally, assays must meet strict standards to be classified as a high-sensitivity cardiac troponin assay: it must maintain a precision of <10% at the 99% URL and be able to report detectable levels below the 99% URL in >50% of healthy men and women [5]. Sensitive tests are critical as patients require prompt medical attention with confirmed diagnosis; any ambiguity may result in missed opportunities during a narrow golden window period.

Both high-sensitivity cardiac troponin T (hs-cTnT) and I assays can be used for the purposes of the ESC algorithms. However, there are important differences between the two markers, for example, in patients with skeletal muscle disorders, it is possible for patients to have fairly normal troponin I with a higher hs-cTnT even in the absence of cardiac disease, for example, in noninflammatory myopathies/myositis/myasthenic syndrome (hs-cTnT can be up to 16 ng/L higher in patients with muscular complaints versus control subjects) [6], neuromuscular disease such as amyotrophic lateral sclerosis [7] and rhabdomyolysis [8]. Hs-cTnT has also been shown to increase with decreasing kidney function, with one study [9] reporting that hs-cTnT baselines increased by 8.2% for every decrease of 5 mL/min/1.73 m^2^ in the estimated glomerular filtration rate (eGFR), although increasing or elevated hs-cTnT was associated with increased morbidity and mortality [10]. Troponin I is less affected by chronic kidney disease (CKD) to some extent, with some studies showing that the ROC area-under-curve for troponin I only decreases from 0.964 to 0.946 in CKD patients [11]. Indeed, a systemic review reported that in patients with end-stage renal disease on hemodialysis or peritoneal dialysis, elevations in hs-cTnI were only reported in 3–67.4%, but up to 39–98% for hs-cTnT [12]. On the other hand, the occurrence of macrotroponin interference is more likely with troponin I than hs-cTnT assays, with some studies reporting that 52.7% (99/188) community patients with elevated hs-cTnI had macrotroponin I [13]. The Roche Elecsys hs-cTnT assay also has a short assay time of 9 min, in contrast to longer assay times for troponin I assays depending on the manufacturer (e.g., Abbott Alinity troponin I is 18 min, Beckman Access hs-TnI is 15 min, and the Siemens Atellica hs-TnI is 10 min).

The use of hs-cTnT in the ESC algorithms has only been established for the Roche Elecsys analyzers. The performance of the Sysmex hs-cTnT assay, performed on their Highly Integrated Single-Cartridge Luminescence Immunoassay System (HISCL), has only been previously reported [14] in a Sysmex internal document (2018), with an excellent CV of 10% at the limit of quantitation of 5 ng/L, and an ROC curve AUC of 0.946 at a cut-off value of 91 ng/L. While there are other published recent HISCL studies, e.g., carcinoembryonic antigen [15] and pre-sepsin [16] assays, the HISCL hs-cTnT assay has not undergone any further validations. In this study, we sought to compare the performance of the new HISCL hs-cTnT assay against the existing Roche Elecsys hs-cTnT assay. In addition, we also established the 99% URL in a representative cohort to facilitate clinical interpretations of results with gender specific subclassifications. We also assessed the influence of renal dysfunction (using the eGFR) on the HISCL hs-cTnT assay within an Asian cohort.

## 2. Materials and Methods

### 2.1. Assay Evaluation

The Sysmex HISCL hs-cTnT was assessed on the HISCL-5000 automated immunoassay analyzer (Sysmex Corp., Kobe, Japan). This is a 1-step sandwich assay where mouse anti-TnT-coated magnetic particles react with the sample hs-cTnT. Thereafter, alkaline-phosphatase-labelled monoclonal mouse anti-TnT reacts with the particle-bound hs-cTnT. Following removal of unbound magnetic particles, a chemiluminescent substrate (chloro-dioxetane phenyl phosphate) is added and a luminescent signal generated which is proportional to the sample hs-cTnT concentration. The new HISCL hs-cTnT assay has a claimed measurement range of 2–10,000 ng/L, an accuracy of ± 20% at low (50–200 ng/L), medium (500–1000 ng/L) and high (5000–10,000 ng/L) hs-cTnT levels with a reproducibility of <10% CV at each level, a reference cut off of <16 ng/L, and a claimed limit of quantitation of 1.5 ng/L (internal company data). The HISCL assay utilizes 6 levels of calibrators (HISCL troponin T hs C0 to C5, C1-C5 contain human serum, and a shelf life of 90 days post-reconstitution), and calibration curves are valid for 30 days. Control materials are provided in the assay kit. The HISCL assay has an assay time of 17 min.

For assay evaluation, anonymized, deidentified leftover sera was stored at −70 degrees Celsius, if not immediately analyzed. The limit of blank and limit of detection were conducted according to recommendations from the Clinical and Laboratory Standards Institute (CLSI) EP17-A2 Guidelines [17]. The limits of quantitation were determined using HISCL diluent (Sysmex Corp., Kobe, Japan) to serially dilute HISCL control reagents (Sysmex Corp., Kobe, Japan) to obtain several testing points. Each was subsequently tested 20 times to obtain a mean value with the associated coefficient of variance (CV%). We curve-fit the results to determine the functional sensitivity at a CV% of 20% and 10%, respectively [18]. Precision was assessed using 2 levels of HISCL control material run 5 times daily over 5 days as per CLSI EP05-A3 guidelines [19]. For the method comparison, samples were analyzed on both the Sysmex HISCL and Roche Elecsys analyzers for hs-cTnT and subsequently compared using Passing–Bablok Regression and Bland–Altman analysis. Values less than the claimed limit of quantitation (1.5 ng/L) were reported as 1.5 ng/L.

### 2.2. 99% URL, and Effects of Increasing Renal Dysfunction on the Hs-cTnT

A total of 1004 healthy individuals over 18 years of age with no known history of hypertension, diabetes and an eGFR ≥ 60 mL/min/1.73 m^2^ were included. Creatinine was assessed using the Roche Elecsys creatinine assay and the eGFR calculated using the CKD-EPI equation [20]. Subjects with cardiac, muscle, liver, renal and pulmonary diseases were excluded. A further 303 samples from subjects with CKD (eGFR < 60 mL/min/1.73 m^2^) were additionally tested to observe the effect of decreasing renal function on the HISCL hs-cTnT. All evaluations were blinded to the analyzer operator.

### 2.3. Statistical Analysis

Data were presented as either the mean ± standard deviation or median (inter-quartile range) where appropriate. No indeterminate or missing results were used. Passing–Bablok regression analysis was also performed to assess the agreement between HISCL and Roche hs-cTnT. Bias was evaluated using the Bland–Altman method. We used MedCalc Statistical Software (version 20.008, MedCalc Software Ltd., Ostend, Belgium) for statistical analyses. For limit of quantitation analysis, we used a non-linear regression model using GraphPad Prism (version 9.2.0, GraphPad Software, San Diego, CA, USA). As this was part of routine clinical laboratory method evaluation, national regulations exempt such investigations from institutional review board (IRB) review. This study was conducted in compliance with STARD guidelines (see Supplementary Table S1).

## 3. Results

### 3.1. Assay Evaluation

**Figure 1:**
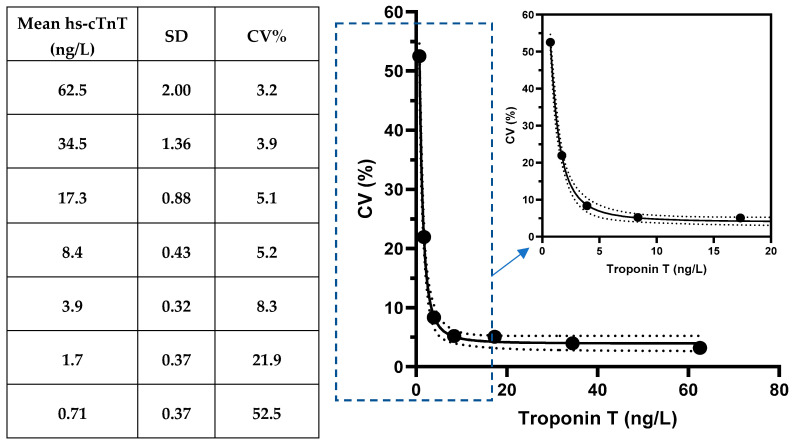
Determination of functional sensitivity with serial dilutions.

The HISCL assay limit of blank was 1.3 ng/L and the limit of detection was determined to be 1.9 ng/L. From our dilution study, hs-cTnT concentrations corresponding to 20% and 10% CV% were 1.8 ng/L and 3.3 ng/L, respectively (see Figure 1). Assay precision for the high-level control was 2.2% (mean 3253 ng/L, SD 70.6 ng/L) and low-level control was 2.5% (mean 106 ng/L, SD: 2.7 ng/L), respectively.

**Figure 2:**
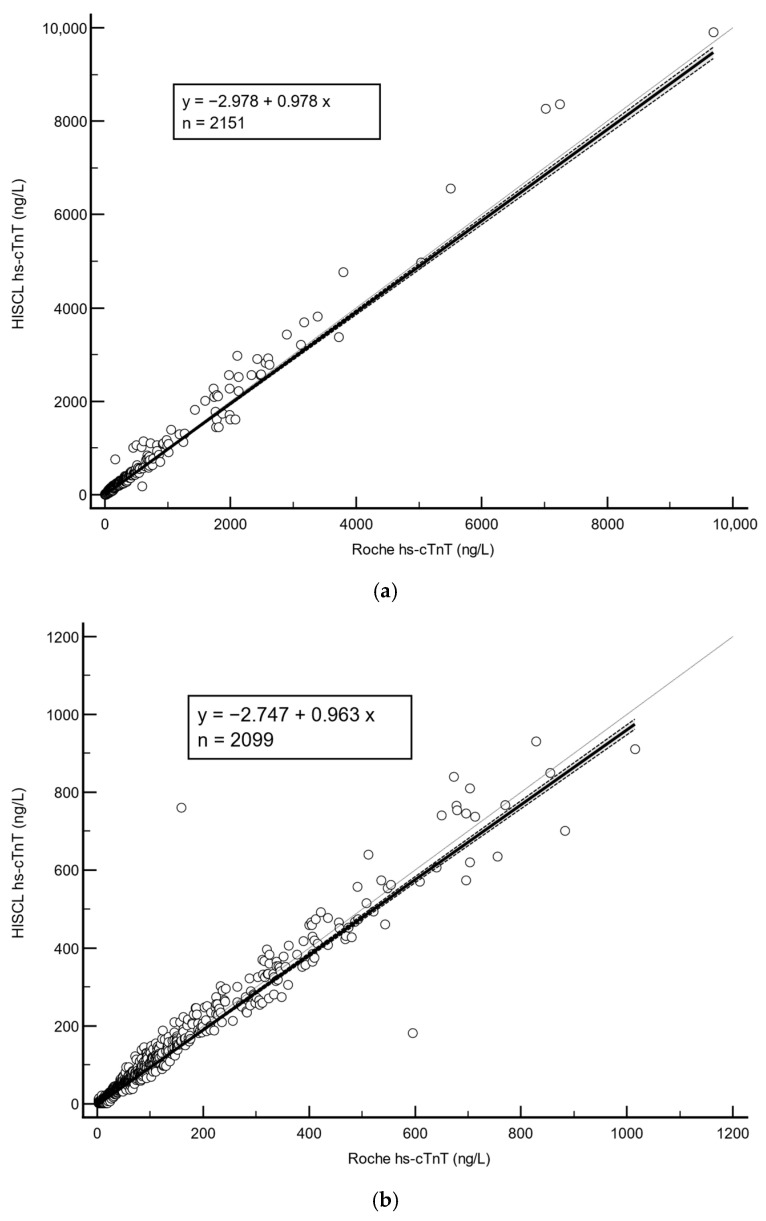

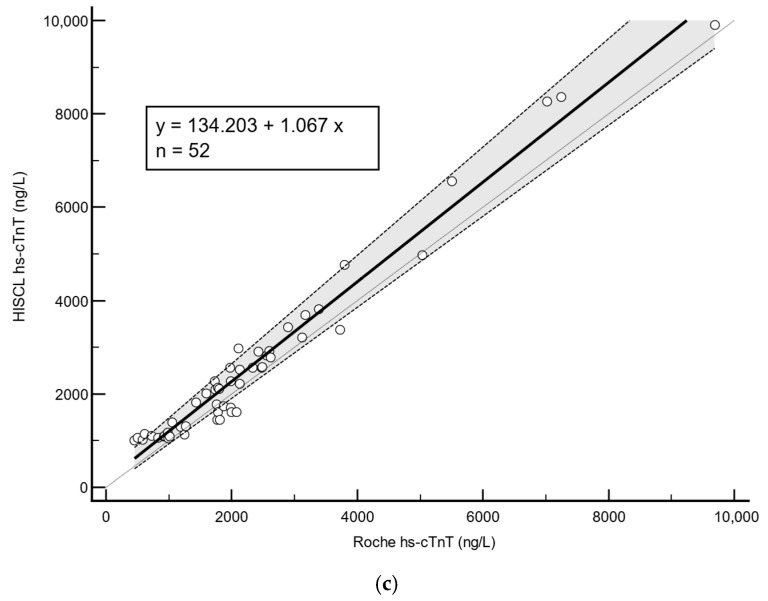
(**a**) Overall Passing–Bablok regression analysis of the Roche vs. HISCL hs-cTnT, (**b**) Passing–Bablok regression analysis of samples with Roche hs-cTnT < 1000 ng/L, and (**c**) Passing–Bablok regression analysis of samples with Roche hs-cTnT > 1000 ng/L.

**Figure 3:**
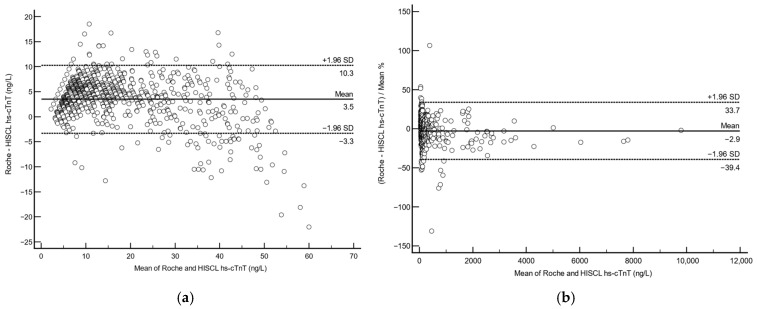
Bland–Altman analysis between HISCL and Roche hs-cTnT, for (**a**) samples with Roche hs-cTnT ≤ 52 ng/L, and (**b**) samples with Roche hs-cTnT > 52 ng/L.

For method comparison between the Roche/HISCL hs-cTnT, we used anonymized leftover samples (*n* = 2151). For these samples, the Roche hs-cTnT ranged from 3.0 to 9689 ng/L while the HISCL hs-cTnT values ranged from 1.5 to 9901 ng/L. Linear regression analysis showed a good agreement between the two assays with an overall Spearman rank correlation coefficient of r = 0.95 (95% CI 0.95–0.96, *p* < 0.0001) (see Figure 2a). For samples with Roche hs-cTnT < 1000 ng/L (*n* = 2099), the correlation coefficient was r = 0.95 (95% CI 0.946–0.954, *p* < 0.0001) (see Figure 2b); for samples with Roche hs-cTnT > 1000 ng/L (*n* = 52) the correlation coefficient was also r = 0.95 (95% CI 0.91–0.97, *p* < 0.0001) (see Figure 2c). As ESC 0/1-h algorithms utilize a cut-off of Roche hs-cTnT > 52 ng/L to rule-in ACS, we used the same cut-off to divide our results into two groups to perform Bland–Altman analysis to see how comparable they were. Bland–Altman analysis showed that for samples with a Roche hs-cTnT ≤ 52 ng/L (*n* = 1692), there was a mean absolute difference of 3.5 ng/L (95% CI 3.0–3.6 ng/L, *p* < 0.0001) (see Figure 3a). For samples with a Roche hs-cTnT > 52 ng/L (*n* = 459), the mean Roche/HISCL difference was 2.8% (95% CI 1.2–4.6%, *p* = 0.0011) (see Figure 3b).

### 3.2. Establishing the 99% URL and High-Sensitivity Status

The dataset analyzed in this study comprised 1004 donor specimens (503 males, 501 females) (see Table 1) consisting of multiple ethnicities with a relatively uniform spread across different age groups. The overall 99% URL was established to be 14.4 ng/L within this group (see Table 2), with gender-specific cut-offs of 17.0 ng/L and 13.9 ng/L for male and female subjects. The corresponding CV% at the 99% URLs were estimated to be 4.34% (overall), 4.23% (males), and 4.37% (females), respectively. More than 50% of hs-cTnT were above the assay limit of detection for both male and female subgroups. Thus, our findings confirm that the HISCL cardiac troponin T test met the criteria defined for a high-sensitivity troponin assay.

Table 1 also presents a further analysis of the distribution of troponin T values within the healthy cohort, with the data split across different age bands. We observed increasing mean and median values among these healthy donors across the entire cohort and within gender subclassifications. Additionally, male donors within the same age band had higher troponin T values than female subjects. To be representative of chest pain patients presenting to our emergency department (typically of older age), we analyzed a subgroup of subjects above 30 years of age (*n* = 849). The calculated 99th-percentile levels using non-parametric analysis in this group were 14.7 ng/L overall, 18.4 ng/L for males, and 13.8 ng/L for females. The proportion of detectable hs-cTnT was 62/69/55% for overall/males/female categories in subjects >30 years old.

### 3.3. The Influence of Renal Dysfunction

To study the effects of renal dysfunction on the HISCL hs-cTnT assay, results were correlated with subjects by CKD stage (subjects with eGFR <60 mL/min/1.73 m^2^: Males/Females 152/151, age 37–99 years old, age median 77 years old, CKD-EPI eGFR range 2–59 mL/min/1.73 m^2^, no subjects on dialysis yet). We observed a general increase in hs-cTnT with increasing CKD stages (G3a, G3b/G4, G5) (Table 3). G5 mean eGFR was nearly 9× higher than the G3a eGFR.

## 4. Discussion

Troponins continue to be one of the most essential tests provided by the clinical laboratory, and our evaluation of the HISCL hs-cTnT shows that it has good agreement with the existing Roche assay and fulfils the necessary criteria to be considered as a hs-cTn assay (CV% at 99% URL < 10%, and hs-cTnT value detectable in >50% in healthy males and females above the limit of detection). Similar to the Roche, the HISCL hs-cTnT 99% URL is higher for males than females, with an increasing mean troponin value with decreasing eGFR. Pending larger studies with outcomes, the Roche hs-cTnT cut-off can probably be applied to the HISCL hs-cTnT for rule-in/out of myocardial injury.

Hs-cTns are essential to the diagnosis of critical cardiac events, as the ability to differentiate non-cardiac and benign diseases among these patients will greatly alleviate the stress and burden on both the emergency department and laboratory services in the hospital. In fact, a significant proportion of the patients with dyspnea and chest pain do not have these critical conditions [21], and ESC 0/1-h and 0/2-h algorithms using hs-cTns have been demonstrated to achieve remarkable accuracy in diagnosis, with the 0/2-h algorithm having an overall accuracy of 89.0% and the 0/1-h algorithm 88.6% in an emergency center population [22]. Indeed, hs-cTnT was able to rate chronic myocardial injury 5 times more than hs-cTnI (20% vs. 4%) while still having similar performance in identifying acute myocardial injury (hs-cTnT 14% and hs-cTnI 15%), with a greater prognostic value for all-cause mortality, incident MI, revascularization, or hospitalization due to heart failure in the convalescent period after an episode of acute chest pain (hs-cTnT/I HR 1.38 vs. 1.14) [23] in a population of 48–73 year old patients.

In this current study, we have evaluated the new Sysmex HISCL hs-cTnT assay for its analytical performance, established the healthy population 99th percentile and evaluated the impact of renal dysfunction on hs-cTnT so that clinical interpretations can be made with greater clarity. Indeed, we have derived the hs-cTnT 99% URL from a cohort that closely follows recommendations for the selection of an appropriate reference cohort by the International Federation of Clinical Chemistry Committee on Clinical Application of Cardiac Bio-Markers [24]. Basic assay evaluation in this work conforms to the standards of CLSI recommendations, and results demonstrated a CV% of <10% at the gender specific 99% URL. In healthy males and females, troponin levels above limit of detection were seen in greater than 50% of subjects. Thus, the HISCL assay qualifies as a high-sensitivity assay as defined by the international federation of clinical chemistry guidelines [25]. Our results also indicated that HISCL hs-cTnT has a slightly better limit of blank and detection than the published Roche Cobas assay. This may be due to the additional wash step to remove non-specific binding in the HISCL immunoassay. The overall HISCL 99% URL (14.4 ng/L) is close to the Roche current package insert (14 ng/L) and similarly show higher hs-cTnT values for males over females. A direct method comparison analysis showed excellent agreement between both methods (r = 0.95), with a mean absolute difference of 3.5 ng/L at Roche hs-cTnT values ≤ 52 ng/L, and 2.8% at Roche hs-cTnT values > 52 ng/L. Even in subjects who were divided between Roche hs-cTnT values < 1000 and >1000 ng/L, the agreement remained close.

In addition, we noted the effects of age and gender on hs-cTnT in our healthy subjects. Our results are consistent with others that have shown that cardiac troponin increases with age [26,27]. In this sub-analysis, we observed mean values for healthy donors above 70 years old to be approximately 3.9-fold higher than the younger cohort (<30 years old). The results indicate that age needs to be factored in when interpreting hs-cTnT results. Using an older population to generate the 99% URL is significant because it ensures that the reference limits are appropriate for patients presenting with chest pain are typically older and at higher risk of myocardial infarction. Indeed, the use of younger patients <40 years old may skew the 99% URL to be lower than expected, leading to over diagnosis of myocardial injury in those >40 years old. This may present an issue when the majority of patients who present with cardiac disease, as shown by population studies in the United Kingdom (*n* = 1,650,052) that cardiovascular disease usually occurs between 65 to 80 years old, with almost all ACS occurring after 40 years old [28]. In Singapore, the overall median age of onset of ACS is 71.4 years old in 2022 [29]. Indeed, specificity of the 99% URL in diagnosing acute disease has been shown to decrease from 98.3% to 82.6% between adults <50 and >75 years old [30], with an associated reduction in positive predictive value. Other studies [31] have also found that there can be significant difference between hs-cTnT 99% URLs between age groups (<50 years 15.8 ng/L, and >70 years old 37.6 ng/L), with 25.6% of patients >70 years having baseline troponins above manufacturer recommended values. Furthermore, hs-cTnT was consistently lower in females than males, regardless of age-group. This is supported in other studies with larger populations that demonstrate that in subjects with no incident cardiovascular events, women have lower hs-cTnT than men (females vs. males ≤ 3.0 vs. 4.5 ng/L) but was more strongly associated with cardiovascular outcomes in women [32]. These lower hs-cTnT values in females is due to their smaller cardiac mass [33]. Thus, future guidelines should take age and gender into account when deriving 99% URLs.

It is also known that lower eGFR is associated with higher cardiac troponin values [33,34], with every 10 mL/min/1.73 m^2^ decrease in eGFR from 90 mL/min/1.73 m^2^ associated with a stepwise increase in hs-cTnT concentrations. Our current investigation is generally consistent with these findings. Indeed, the mean value for G3a cohort (36 ng/L) exceeded that of our established healthy 99th percentile. This will have direct implications on hs-cTnT interpretation in chest pain evaluation and special care needs to be accorded to patients with CKD stage 3 and beyond. Some studies have shown that up to 43% of CKD patients can have baseline values above the conventional 99% URL, increasing to up to 68% in those with eGFR < 30 mL/min/1.73 m^2^ [35], and larger studies would be required to provide guidelines on how best to use hs-cTnT for the identification of ACS in CKD patients. The adoption of optimized cut-offs with serial testing algorithms may be helpful in CKD patients, as shown in one study [11] that adjusted diagnostic cut-offs for CKD patients to match the specificity of hs-cTnT in non-CKD patients (from 14 to 50 ng/L), and together with an algorithm with serial testing, achieved a diagnostic performance of up to 55%.

Our study shows good agreement between the two hs-cTnT assays. However, there remains more variation between hs-cTnI assays [36]. ESC guidelines have different cut-offs for different assays [1], and even in general population studies there are biases between hs-cTnI assays despite good correlations (r of 0.61–0.70 between Abbott/Siemens/Ortho, with percentage discordant findings of up to 7.5% in subjects above the Ortho hs-cTnI 99th percentile) [37]. Variation between assays is also being increasingly recognized at low concentrations near important decision levels, with varying precision performance between assays [38,39]. This continues to cause confusion in the interpretation of hs-cTnI between platforms. For hs-cTnT ESC rule-out/rule-in protocols have cut-offs only for the Roche hs-cTnT Elecsys platform. Although not yet included in the ESC guidelines, the HISCL assay can probably use the same Roche ESC cut-offs given its close agreement with the Roche assay. It is thus preferable to use the same platform in serial troponin testing, as there may be significant categorization differences between different assays.

A limitation of our study is that it is a single-center assessment and may not be generalizable to other populations, although we did include various ethnic groups. Until today the only the Roche hs-cTnT assay is validated for the ESC ACS rule-out/rule-in protocols [1], and further larger studies would be required to refine appropriate cut-offs for the HISCL hs-cTnT assays. A limitation of the HISCL assay is the non-provision of control materials with lower hs-cTnT concentrations, and we have highlighted this to the manufacturer. In fact, the IFCC expert committee recommends that control materials should have values that are between the limit of detection and the female 99% URL, higher than but close to the male 99% URL, and that challenges the upper analytical range would be needed [40].

## 5. Conclusions

In conclusion, the Sysmex HISCL hs-cTnT has satisfactory performance, and fulfils the criteria for a hs-cTn assay, with specific 99th URLs for males and females. Expectedly, the baseline Sysmex hs-cTnT increases with age and decreasing eGFR, and caution must be exercised when interpreting hs-cTnT in older patients or patients with CKD.

## Figures and Tables

**Table 1:** Demographics of subjects used for assessment of 99% URL.

**Total Population, *n***	1004		
**Gender, *n* (%)**	Male		503 (50.1)
	Female		501 (49.9)
**Age, range (Median)**	18–94 (49)		
**Ethnic Origin, *n* (%)**	Chinese		436 (43.4)
	Malay		149 (14.8)
	Indian		103 (10.3)
	Eurasian		2 (0.20)
	Other/Unknown		314 (31.3)
**Total Population Age Groups**	***n***	**Hs-cTnT Mean (ng/L)**	**Hs-cTnT Median (ng/L)**
<30	155	1.6	0.9
30–39	189	1.7	0.9
40–49	161	2.8	1.8
50–59	169	4.1	3.0
60–69	164	4.6	3.9
≥70	166	6.3	5.7
**Males Age Groups**			
<30	80	2.0	1.3
30–39	85	2.7	1.7
40–49	85	4.0	2.5
50–59	93	4.7	3.6
60–69	97	5.8	5.0
≥70	63	8.2	7.5
**Females Age Groups**			
<30	75	1.2	0.1
30–39	104	0.9	0.0
40–49	76	1.5	0.9
50–59	76	3.4	2.1
60–69	67	2.9	2.1
≥70	103	5.1	4.5

Abbreviations: Hs-cTnT: high-sensitivity cardiac troponin T.

**Table 2:** Performance of the 99% URL for the HISCL hs-cTnT assay.

99th Percentile (ng/L)	%CV at 99th Percentile	Percentile Normal Measured ≥ Limit of Detection
Overall: 14.4 F: 13.9, M: 17.0	Overall: 4.34% M/F: 4.23%/4.37%	Overall: 56.6% M/F: 63.0%/50.1%

**Table 3:** Influence of eGFR on HISCL hs-cTnT measurements (GFR grading reference from KDIGO classifications).

KDIGO CKD Stage	*n*	HISCL Hs-cTnT (Range, Mean) (ng/L)
G3a	110	1.5–453, 36
G3b	85	2.8–3214, 187
G4	41	2.6–1617, 152
G5	67	10.7–1781.38, 319

## Data Availability

The datasets generated during and/or analyzed during the current study are not publicly available due to privacy issues and national laws but are available from the corresponding author on reasonable request under the provision that data may not leave the hospital/center premises.

## Supplementary Materials

The following supporting information can be downloaded at: https://www.mdpi.com/article/10.3390/diagnostics15151838/s1, Table S1: STARD checklist.

## Abbreviations

The following abbreviations are used in this manuscript:

ACS	acute coronary syndrome
ESC	European Society of Cardiology
99% URL	99th-percentile upper reference limit
Hs-cTnT	high-sensitivity cardiac troponin T
eGFR	estimated glomerular filtration rate
CKD	chronic kidney disease
HISCL	Highly Integrated Cartridge Luminescence Immunoassay System
CV%	coefficient of variance
